# Interposition Arthroplasty in Untreated Chronic Dislocation of the Elbow

**DOI:** 10.5435/JAAOSGlobal-D-21-00034

**Published:** 2022-05-09

**Authors:** Gustavo Armando Tafoya-Arreguín, José Rene Castillo-González, Irydia-Guadalupe Pellegrini-Verduzco, José de Jesús Martínez-Ruíz, Rubén Daniel Esqueda-Godoy, Jorge Iván Arce-Rosas

**Affiliations:** From the Department of Orthopaedics and Traumatology, the University Center of Health Sciences, University of Guadalajara “Benemerito Antiguo Hospital Civil de Guadalajara Fray Antonio Alcalde,” Instituto de Reconstrucción Articular y Medicina Deportiva de Occidente, Guadalajara, Jalisco Mexico (Tafoya-Arreguín); Orthopaedics and Traumatology, the University Center of Health Sciences, University of Guadalajara “Benemerito Antiguo Hospital Civil de Guadalajara Fray Antonio Alcalde,” Guadalajara, Jalisco Mexico (Tafoya-Arreguín, Dr. Castillo-González, and Dr. Pellegrini-Verduzco); the Department of Orthopaedics and Traumatology, the University Center of Health Sciences, University of Guadalajara “Benemerito Antiguo Hospital Civil de Guadalajara Fray Antonio Alcalde,” Guadalajara, Jalisco Mexico (Tafoya-Arreguín, Dr. Martínez-Ruíz, and Dr. Arce-Rosas); the Department of Orthopaedics and Traumatology, Hospital Regional de Tepatitlán, Tepatitlán de Morelos, Jalisco, Mexico (Tafoya-Arreguín, Dr. Esqueda-Godoy); and the Instituto de Reconstrucción Articular y Medicina Deportiva de Occidente, Guadalajara, Jalisco, Mexico (Tafoya-Arreguín).

## Abstract

To describe the clinical outcomes of interposition arthroplasty with transposition of the medial epicondyle to the coronoid process and articulated external fixation in patients with untreated chronic dislocation of the elbow.

**Methods:**

Fourteen consecutive patients diagnosed with untreated chronic elbow dislocation performed a complete radiological and physical examination. The same surgeon treated all patients with a same technique. Passive mobilization started immediately in addition to the vigorous care of the surgical wound and surrounding skin.

**Results:**

A total of 14 patients were treated, with a mean age of 31 years, with the nondominant side being the most affected (65%). In the immediate postoperative period, the initial Mayo Elbow Performance Score was 60 pts. In all cases, the distraction from the articulated fixator was removed, and there was an average of 16 pts improvement at the time of removal. A hinged elbow orthosis was placed for 4 weeks starting strengthening and obtained radiographic integration of the neocoronoids; ranges of motion of flexion 122°, extension −6°, and pronosupination 70°, without data of any direction instability.

**Conclusion:**

Considering that this is one of the longest series with a follow-up of more than 60 months of evolution in our patients, the result is completely satisfactory, achieving the objective of a minimum range of motion of 100° in addition to elbow stability.

Biomechanically, the elbow is considered one of the most complex joints because of the synergistic need for four joints^1^ (humerus-ulna, proximal radioulnar, radiocapital, and distal radioulnar) to meet its daily life functional objective; the minimum requirement of range of motion being 100° in flexion-extension and 40° in pronosupination, while remaining stable throughout the range of motion.^2^

Elbow dislocation represents 20% of upper extremity dislocations, followed by the shoulder joint^3^; the radius of complications is given by the poor diagnostic and therapeutic approach because most of the patients received primary care in rural areas whose first contact was a family doctor and their proper assessment was delayed, this being the cause of the persistence of the lesion.^3,4^

The chronicity of the injury is defined by its persistence of longer than 3 weeks^5^; this entails a change in the anatomical structures of the joint that prevent its reduction, in addition to cyclical damage to the ligament and cartilaginous structure of the elbow.^3,6,7^

Elbow instability associated with bone structures in chronic dislocations ranges from 30% for fractures of the coronoid process and 40% for radial head fractures.^8^ The rest are adaptive findings such as triceps contracture, involvement of the ligament complex, probability of injury to the ulnar nerve, contracture of the joint capsule, and joint fibrosis, among others.^8-10^

Interposition arthroplasty was initially described for the management of primary and posttraumatic osteoarthritis of the elbow in young patients; some reports in the literature, however, support its use,^11,12^ coupled with distraction external fixation,^10^ as treatment in young patients with untreated chronic dislocation of the elbow due to cartilage loss.

## Methods

**Figure 1:**
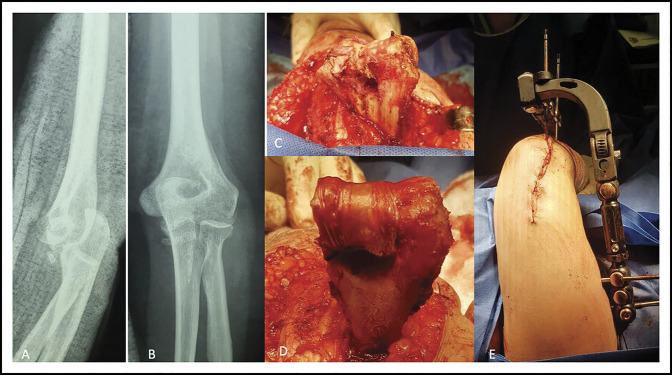
**A** and **B**, Presurgical AP and lateral radiographic view of our patient. **C**, Trans-surgical images showing abundant chondral lesion. **D**, Placement of interposition arthroplasty. **E**, Articulated external fixation with cutaneous closure.

An interposition arthroplasty was performed with neocoronoids in 14 consecutive patients diagnosed with a chronic elbow dislocation for longer than 6 months, previously diagnosed using multislice tomography study and radiographic series (AP, lateral, and oblique). Measurements of lesion in the coronoid process (greater sigmoid notch) and radial head (neck of the radius angle) were taken by the same surgeon (Figure 1).

All patients underwent functional assessment and scales (Mayo Elbow Performance Score [MEPS] and QuickDASH) initially and during their follow-up to document their evolution, in addition to clinical measurement of ranges of motion and varus-valgus and AP stability tests, as well as visual analog pain scale (0 to 10 line). In addition, a radiographic evaluation to measure the olecranon notch angle was performed.

### Surgical Technique

**Figure 2:**
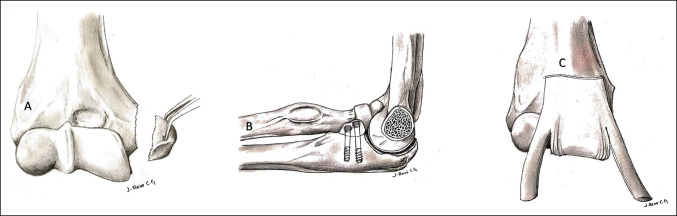
**A** and **B**, Diagram of taking epitrochlear neocoronoids and their fixation; (**C**) graft placement and fixation for interposition with flaps.

All patients were treated by the same surgeon; under general anesthesia plus regional analgesia (supraclavicular block), ischemia was not found in any case; the patient was in a supine position, posterior approach with triceps split, transposition of the ulnar nerve, capsular release, medial epicondyle osteotomy, and transposition to neocoronoids (because of the medial epicondyle has similar anatomical characteristics to the coronoid as measured by the topographic study and the polyhedral conformation that width and height resembles the coronoid from the base of the olecranon joint surface), fixed with two headless compression screws (Figure 2).

Subsequently, in all patients, trochlear and capitellar joint area débridement was performed with the removal of nonviable cartilage to subsequently take a semitendinosus graft from the contralateral leg to carry out interposition arthroplasty, performing multiple fenestration to the distal humerus and tunneling the ulna to achieve stability with flap (lateral and medial) of the same tendon graft, fixing it in a transosseous manner and with No. 2 nonabsorbable high-resistance sutures.

Afterward, reduction and placement of articulated external fixation with humeroulnar distraction was performed, before a transoperative assessment of AP and varus-valgus stability. Finally, the triceps is closed in block with continuous suture anchored both medially and laterally of the flexor and extensor mass of the wrist, respectively, with a No. 2 high-resistance nonabsorbable suture, in addition to transposition of the ulnar nerve and finally closure by planes of subcutaneous cell and skin tissue.

### Postoperative

The immediate hospital postoperative management plan was performed based on free movements of the hand and wrist; self-passive and passively assisted to elbow in flexion, extension, pronation, and supination to tolerance; physical means (topical ice 15 minutes every 3 hours), antiedema bandage, and IV analgesia alternating paracetamol 1g IV/ketorolac 30 mg IV every 6 and 8 hours, respectively.

At the time of their hospital discharge, they continued with physical means (topical ice 15 minutes every 3 hours for 3 or more days), oral analgesia for pain control (ketorolac 10 mg/paracetamol 750 mg every 8 and 6 hours, respectively), and antibiotic therapy in the double scheme (clindamycin/ciprofloxacin for 10 and 7 days, respectively, according to the local clinical practice guide), in addition to oral celecoxib 200 mg every 24 hours for 4 weeks as prophylaxis against the possibility of heterotopic ossification.^13,14^

### Follow-up

Follow-up was performed for 15 days for the removal of stitches and performed at 12 weeks for radiographic control and removal of distraction in external fixation; at 14 weeks, in all cases, the external fixator was removed, followed by the placement of the Mayo articulated orthosis with freedom of movement. A workplan in the rehabilitation clinic to improve ranges of motion and follow-up every 4 weeks until discharge was incorporated.

Ranges of mobility, AP stability, and varus-valgus stability were clinically assessed, whereas the olecranon notch angle and joint congruence were measured radiometrically to compare with the initial measure and evaluate the articular contention in addition to monthly functional scales until discharge at 32 weeks.

For data analysis, descriptive statistics were performed with measures of central and inferential tendency with comparison of means and correlations in intervening variables.

## Results

A total of 14 patients, seven males and seven females, with a mean age of 30 years (17 to 40 years), with a diagnosis of chronic elbow dislocation was included with a follow-up of, on average, 19 months of evolution (12 to 32), and being the dominant limb in 35% (5) of the cases (Table 1).

**Table 1:** General Description of All Patients

Patient Description	Sex	Age	Evolution	Notch Pre (°)	Notch Post (°)	Flex (°)	Ext (°)	Pron (°)	Sup (°)	Follow-up	MEPS	QuickDASH
1	M	36	22	8	32	124	−8	54	70	50	100	2, 3
2	M	31	12	10	36	120	−6	52	70	48	100	0
3	F	28	14	10	37	122	−6	50	70	26	95	4, 5
4	M	33	16	8	38	110	−10	52	70	60	85	4, 5
5	F	31	12	6	36	128	−8	58	70	33	100	2, 3
6	F	17	14	8	32	116	−14	50	60	28	100	15, 9
7	F	40	32	10	34	122	−18	48	70	48	85	2, 3
8	M	26	20	8	30	120	−2	50	70	60	100	6, 8
9	F	33	27	8	33	124	−4	52	60	54	100	0
10	F	29	19	8	30	126	−6	54	60	32	100	4, 5
11	M	28	14	6	31	124	−8	56	70	20	95	11, 4
12	M	21	27	10	37	112	−4	48	70	18	100	2, 3
13	M	28	24	8	30	108	−10	58	60	41	80	6, 8
14	F	35	13	10	37	128	−14	60	60	24	95	6, 8

Radiometric evaluation initially showed an increase in the olecranon notch angle with an initial mean of 8.4° (6° to 10°) to 33.8° (30° to 38°) at the end of follow-up (*P* < 0.001; 95% CI, 32 to 35). The measure to evaluate the articular congruence was the angle of the sigmoid cavity of the ulna with a mean of 43° (42° to 44°). The mean follow-up was 38.7 months, with a minimum of 18 months and a maximum follow-up of 60 months.

The clinical functionality and functional scales were compared with the initial measurements, showing that initial MEPS average of 29.3 pts (20 to 35) and at follow-up was 95.36 pts with a range of 80 to 100 pts (*P* < 0.001; 95% CI, 91 to 99).

When assessing the initial QuickDASH scale of 72.9% and at the end of the follow-up, a mean of 5% was observed with a range of zero to 15.9% (*P* = 0.001; 95% CI, 2.5 to 7.5).

**Figure 3:**
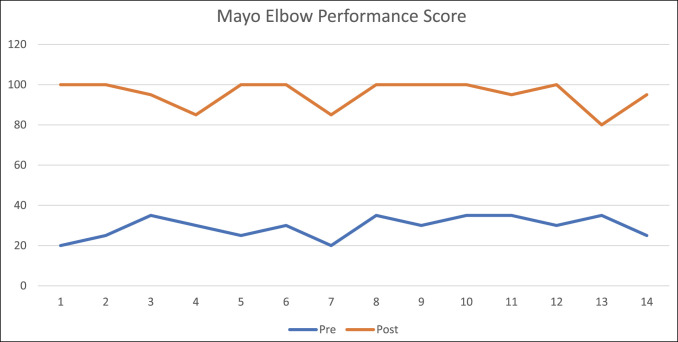
Graph showing the Mayo Elbow Performance Score (MEPS) in each patient.

**Figure 4:**
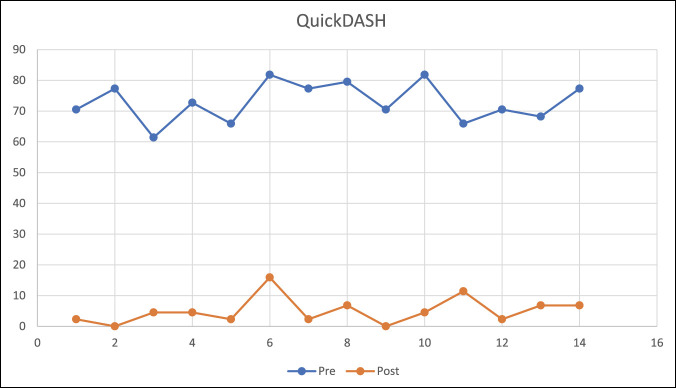
Graph showing the QuickDASH before and after treatment.

Clinically, the final ranges of motion had an average of extension −8.43°; flexion 120.29°; 53° pronation; and 66.43° supination; the visual analog pain scale decreased from an initial of 7 pts (4 to 8 pts) to a mean of 1.93 (zero to 5) (*P* = 0.001; 95% CI, 1.5 to 2.7; Figures 3 and 4).

When contrasting the variables of evolution time with the final result, there was no statistical significance with *P* = 0.497 assessed with the QuickDASH, whereas the MEPS significance with the evolution time was *P* = 0.008.

There were no major or minor complications associated with the external fixator in our group.

Finally, when performing the correlation analysis between variables (sex, age, activity, and dominance of the limb), no notable correlations were obtained for the study.

## Summary

In our study cohort, the interposition arthroplasty of the elbow with neocoronoids from an epitrochlea with autograft obtained from the semitendinosus plus articulated external fixation is an adequate surgical technique in patients with untreated chronic dislocation of the elbow both clinically and using functional scales achieving fully optimal functional objectives.

The interposition arthroplasty of the elbow previously reported by Morrey^12^ as treatment of untreated chronic dislocations of the elbow has been a standard for the management of this pathology. Compared with reports in the literature, our series provides us with better results: Naidoo^15^ included a series of 23 patients, 8 (33%) of them reported results with a range of motion less than 60°, 5 patients (20%) with a range of motion between 60° to 90°, and only 10 patients (40%) reported a range greater than 90° of mobility. In this series, it was not mentioned whether joint congruence persisted at follow-up, nor did data on elbow stability. There is no mention of the previous state of the coronoid, whereas in our series, the average range of functional travel was 112°, and no patient presented clinical nor subjective instability.

Arafiles^4^ in his series of 11 patients reported a mean of 105° of movement after a 2-year follow-up. In another series, Mahaisavariya et al^16^ after a 4-year follow-up reported a mean of 82° of mobility range in 24 patients. They also omitted report on data regarding the stability or state of the prior and postsurgical coronoid.

Donohue et al^8^ performed a review and marked stabilization through repair of the lateral and medial complexes as the ideal treatment while protecting with external fixation obtaining good results; however, in their report, the patients did not present extensive chondral lesions.

We are aware that our study presents limitations in terms of the number of cases, as well as not assessing the periarticular muscle strength (biceps, triceps, flexors, and wrist extensors) in an objective manner that is feasible to add to new studies.

**Figure 5:**
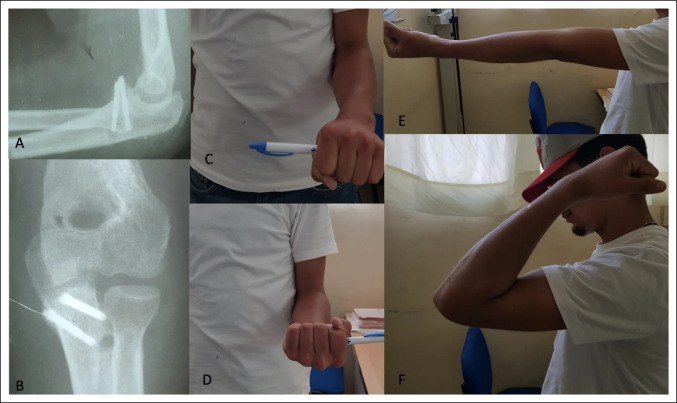
Follow-up at 18 months. **A** and **B**, Postsurgical AP and lateral radiographic view of our patient. **C**–**F**, Range of motion of our patient at the final follow-up.

Our work, compared with what is reported in the literature, presents a substantial contribution by including the epitrochlear neocoronoids and physiotherapy earlier. In addition to evaluating joint congruence, clinical and subjective data of instability, ranges of motion, and stratifying through functional scales provide adequate support for the implementation of this subsequent procedure (Figure 5).

In chronic dislocations, it continues being a challenge to achieve adequate management while preserving stability and ranges of motion.

In our presented series, interposition arthroplasty with neocoronoids performed methodically demonstrated to be an excellent treatment option for patients with untreated chronic dislocation of the elbow.

When compared with other series reported in the literature, the range of motion obtained in our patients complies satisfactorily both clinically and in MEPS and QuickDASH assessments.

A major series and a comparative study are required to demonstrate the effectiveness of neocoronoids in the treatment of untreated chronic dislocation of the elbow.
